# Vascularization Strategies in 3D Cell Culture Models: From Scaffold-Free Models to 3D Bioprinting

**DOI:** 10.3390/ijms232314582

**Published:** 2022-11-23

**Authors:** Shamapto Guha Anthon, Karolina Papera Valente

**Affiliations:** 1 Department of Biomedical Engineering, University of Victoria, Victoria, BC V8W 2Y2, Canada; 2 Department of Mechanical Engineering, University of Victoria, Victoria, BC V8P 5C2, Canada

**Keywords:** vascularization, 3D cell culture, bioprinting, spheroids, scaffold-free, scaffold-based, microfluidics, drug screening, tumor modelling

## Abstract

The discrepancies between the findings in preclinical studies, and in vivo testing and clinical trials have resulted in the gradual decline in drug approval rates over the past decades. Conventional in vitro drug screening platforms employ two-dimensional (2D) cell culture models, which demonstrate inaccurate drug responses by failing to capture the three-dimensional (3D) tissue microenvironment in vivo. Recent advancements in the field of tissue engineering have made possible the creation of 3D cell culture systems that can accurately recapitulate the cell–cell and cell–extracellular matrix interactions, as well as replicate the intricate microarchitectures observed in native tissues. However, the lack of a perfusion system in 3D cell cultures hinders the establishment of the models as potential drug screening platforms. Over the years, multiple techniques have successfully demonstrated vascularization in 3D cell cultures, simulating in vivo-like drug interactions, proposing the use of 3D systems as drug screening platforms to eliminate the deviations between preclinical and in vivo testing. In this review, the basic principles of 3D cell culture systems are briefly introduced, and current research demonstrating the development of vascularization in 3D cell cultures is discussed, with a particular focus on the potential of these models as the future of drug screening platforms.

## 1. Introduction

The development path for a drug to transition from preclinical studies to clinical trials is time consuming and expensive, with a particularly low success rate of less than 10% [1]. While the emergence of newer technologies has evidently accelerated the R&D processes in many sectors, the average number of new drugs approved by the US Food and Drug Administration has declined over the years [1]. While the general likelihood of a drug getting to the market is less than 10%, the success rates for potential anti-cancer drugs drop down to about 7% [1]. One of the main challenges when testing anti-cancer drugs is accurately mimicking the cancer microenvironment. Advancements in the tissue engineering field have made possible the accurate modeling of the tissue microenvironment in vitro and may be the key to overcoming this lack of efficacy in drug development.

Current approaches to anti-cancer drug screening involve using two-dimensional (2D) in vitro systems and checking for endpoint measurements such as cell viability or proliferation [2]. The results are often misleading, as 2D systems are unable to fully capture the biomimetic characteristics observed in a three-dimensional (3D) microenvironment. Observations from cancer models indicate that tumor cells exhibit different morphologies, proliferation and glycolysis rates, and drug sensitivities in 2D cultures compared with a 3D system [3,4,5,6,7]. However, although promising, 3D cell cultures also fail to replicate the dynamic tissue microenvironment due to a lack of fluid flow prefusion [8]. To overcome this limitation, vascularization techniques for 3D cell culture models have primarily been the recent focus of tissue engineering.

The functionality of tissues and organs is dependent on the constant supply of oxygen and nutrients. Vascularization refers to the process of formation of blood vessels within a structure, ensuring cellular proliferation. Vasculature ensures the sufficient supply of oxygen and nutrients to cells, along with the removal of metabolic wastes in vivo by establishing a diffusion gradient. Historically, 3D tissue-engineered structures have been constrained in terms of size and scalability due to the lack of vasculature [9]. The diffusion limit of oxygen limits the thickness of the tissue models between 100 and 200 μm, correlating to the maximum distance among adjacent capillaries in vivo [10,11]. Inadequate supplies of oxygen and nutrients have been observed deeper inside larger tissue constructs, resulting in hypoxia, consequently inhibiting the functionality of the tissue [9,12]. The lack of vascularization also limits the sufficient supply of oxygen and nutrients, and as a result, prevents the maturation of the 3D tissue model due to growth arrest or cell death [13,14]. Vascularization in 3D cell cultures creates added physiological constraints, providing a true replication of the tissue microenvironment by showcasing the cell–cell and cell–extracellular matrix (ECM) interactions, and the dynamic flow conditions, resulting in more accurate response during drug screening [15].

Therefore, vascularization strategies have been increasingly employed in 3D cell culture models in recent years. Vascularization allows 3D cell cultures to behave similarly to cells observed under in vivo conditions and to grow beyond the constraints set by the diffusion limit of oxygen without resulting in any avascular and hypoxic conditions [16]. Creating an accurate replication of the native tissue microenvironment allows researchers to reliable model drug interactions in 3D cell cultures, reducing the current discrepancies observed in in vitro drug testing platforms. Advances in the field of tissue engineering have opened many strategies for achieving vascularization in larger 3D models, with notable successes being observed in using manual assembly, bioprinting, or microfluidic techniques [17,18]. In this review, the basic principles of 3D cell cultures are detailed, and the different techniques currently employed to achieve vascularization in spheroid-based, bioprinting-based, and microfluidic-based 3D cell culture models are discussed, highlighting their impact and limitations and providing insights into the future of 3D cell culture models as drug screening platforms.

## 2. Three-Dimensional Cell Cultures

Two-dimensional cell cultures attempt to mimic native tissues by mapping a cellular monolayer onto a flat, rigid glass or polystyrene surface containing a nutrition medium [19]. However, the set-up fails to capture the in vivo dynamic cellular interactions due to the absence of signals arising from neighboring cells and the ECM [19,20]. This difference in the spatial and temporal organization in 2D cell cultures hinders the accurate growth and development of cells owing to different signal transduction cascades causing cell behaviors different from those of in vivo cells [17,21,22,23,24]. Two-dimensional monolayer cultures are also subjected to a uniform supply of oxygen and nutrients compared with 3D settings, where the primary means of oxygen and nutrient delivery is via diffusion, which varies with depth from the surface [19]. The cells in the 3D culture are, therefore, at different stages of the cell cycle and are a more accurate representation of in vivo cells [19]. The variability of the in vivo tissue microenvironment can also be more accurately captured in 3D cell cultures due to the ability to create “designer” microenvironments in three dimensions, owing to the recent advancements in cell culture techniques and tissue engineering [17,25].

Alexis Carrel reported one of the first uses of 3D cell cultures in 1912, where suspended scattered cells cultured in Ringer’s Solution were observed to produce results identical to those obtained by culturing fragments of tissue [26]. While 3D cell cultures have advanced considerably from the initial set-up, the three basic requirements to mimic native tissue microenvironment still remain the same: cells, nutrient medium, and a material to mimic the extracellular matrix [18]. Therefore, the simplest in vitro 3D cell culture method involves culturing suspended cells in a nutrient medium. The tendency of cells to aggregate in the three dimensions results in the formation of structures called spheroids. Spheroid parameters such as size, shape, density, surface features, and internal textures can be altered by tuning the culture medium or the cell type, matching the tissue microenvironment of interest to predict the outcomes of drug delivery and efficacy studies [27,28]. Alexis Carrel observed that the spheroid models were constrained in volume due to the limited access of nutrients in the core of the spheroids [26]. Developments in the choice of cell lines and the nutrition medium in recent years have allowed researchers to overcome this issue, allowing spheroids to reach sizes of 500 μm or above [27]. However, the cores of spheroids are still devoid of nutrition, resulting in hypoxia and eventually necrosis due to acidic pH and the collection of metabolic wastes [27]. Although this situation is not ideal, these spheroid models can still be used for modeling avascular solid tumors with necrotic cores [27,29,30,31]. 

While multiple techniques have been developed to address the scalability issue in spheroids, the main requirement for an increase in size and complexity is the presence of a scaffold [18]. Three-dimensional cell cultures can, therefore, be categorized under two major groups: scaffold-free and scaffold-based models. The following sections discuss the different approaches towards both scaffold-free and scaffold-based 3D cell culture models.

### 2.1. Scaffold-Free 3D Cell Cultures

**Figure 1:**
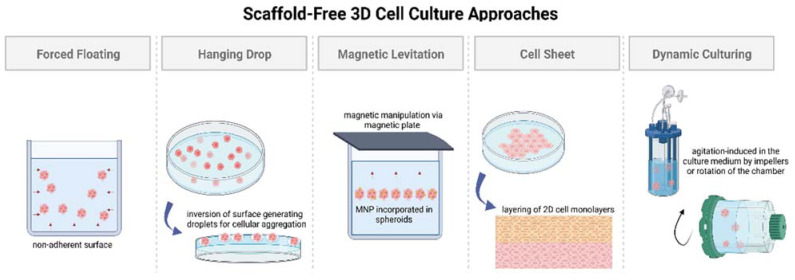
Illustration showing different approaches towards scaffold-free 3D cell culture models. Created with BioRender.com, accessed on 25 September 2022.

Multiple techniques have been developed over the years to culture cells in three dimensions without employing scaffolds, such as forced floating, hanging drop, magnetic levitation, cell sheet, and agitation-based methods. In almost all the techniques, the cells are influenced to aggregate, forming spheroids. The advantages of scaffold-free 3D cell cultures lie in the simplicity of the models, the reproducibility of results, the ease of preparation, scalability, and applicability to various cell lines [32]. Scaffold-free 3D cell cultures can be further categorized into two groups, based on the state of the culture medium: static or dynamic (illustrated in Figure 1). In this review, these two categories are discussed based on the ability of the technique to reproduce a biologically active microenvironment via a stagnant (static) culture medium or a non-stagnant (dynamic) culture medium.

#### 2.1.1. Static 3D Cell Culture

Static 3D cell culture media typically employ non-adherent surfaces to promote spheroid formation via cellular aggregation. The most common methods for culturing cells suspended in a static medium are forced floating, hanging drop, and magnetic levitation [33]. A special case of scaffold-free static medium employing cell sheet engineering principles is also highlighted in this section; the four above-mentioned methods are detailed as follows, and the highlights and limitations for each method have been provided in Table 1:

1. The forced floating method, or liquid overlay method, employs uncoated low-adhesion plates or ultra-low attachment (ULA) plates coated with a hydrophilic polymer [34]. The negatively charged inert polymer coating reduces protein adsorption, inhibiting cellular attachment [35]. The ease of preparation and maintenance of forced floating cultures and the possibility of automation makes it suitable for high-throughput screening [32,34,36]. However, this method encounters issues with the variability of the size and shape of the spheroid models, increasing the difficulty in obtaining reproducible results [33].
2. The hanging drop method involves the inversion of a culture plate containing a cell suspension to create droplets [37,38]. The inversion creates a free liquid–air interface where the micro-adhesive force from the substrate surface is higher than the weight of the accumulated cells in the droplet, resulting in spheroids [34,37,38]. Although this simplistic technique has shown around 90% reproducibility rate in the formation of multicellular tumor spheroids (MCTs), difficulties remain in medium exchange and its application in cell-based assays [32,37,38].
3. Magnetic levitation is a suspension culture technology that aims to address the biodegradability issue surrounding porous scaffold and protein matrices in 3D cell cultures [38,39,40,41]. The technique involves the magnetic manipulation of bioinorganic hydrogel incorporated with magnetic nanoparticles (MNPs), such as magnetic iron oxide (Fe_3_O_4_, magnetite) and gold nanoparticles [39]. Incorporation is achieved via overnight incubation, and the cells are cultured by levitating them using a magnet placed above the plate [37]. Similar to the hanging drop method, magnetic manipulation directs the cells towards the air–liquid interface, taking advantage of the tendency of cells to aggregate and form spheroids. The magnetic levitation method results in spheroid formation within 16 h without requiring a specialized medium [42], making it suitable for high-throughput screening studies.
4. Cell sheet engineering is a tissue-engineering approach towards scaffold-free 3D cell cultures that has showcased safety and efficacy in preclinical and clinical trials for developing implantable devices [43]. The manual gathering of suspended cells into 3D tissues eliminates the issues of gap junctions and unfavorable host responses towards biomaterials as observed in scaffold-based approaches [44]. The cell sheet engineering process involves building 3D tissues by layering 2D cellular monolayers known as “cell sheets” on a surface coated with a temperature-sensitive polymer [44]. The technique offers co-culturing and promotes the development of prevascularized networks through efficient cell–cell and cell–ECM interactions [43]. However, necrosis has still been observed in long-term cell cultures in the middle layers, especially when the tissue constructs go beyond a thickness greater than that of four-layered cell sheets (>100 μm), suggesting the need for vascularization due to insufficient ECM formation [44,45]. Achieving vascularization in cell sheets via co-culturing with endothelial cells (ECs), along with the incorporation of advanced bioreactor systems, is crucial for the creation of thicker cell sheet constructs [45].

**Table 1:** Highlights and limitations of scaffold-free static 3D cell culturing approaches.

Static 3D Culturing Technique	Highlights	Limitations
Forced floating	Ease of preparation and maintenance, high throughput, and suitable for automation	Variability of size and shape of spheroids and difficulty in obtaining reproducible results
Hanging drop	Favorable reproducibility rate	Difficulties in medium exchange and incorporation with cell-based assays
Magnetic levitation	Uniformly shaped spheroids, no specialized medium, and high throughput	Requires specialized equipment and is a time-consuming process
Cell sheet engineering	Promotes prevascularization, and efficient cell–cell and cell–ECM interactions	Necrosis observed in larger constructs and sheets not necessarily replicating the native tissue microarchitecture

#### 2.1.2. Dynamic 3D Cell Culture

Dynamic 3D cell culturing techniques focus on creating controlled, reproducible dynamic flow conditions to mimic the tissue microenvironment in vivo.

Popular scaffold-free dynamic 3D cell culture techniques employ agitation-based approaches by means of bioreactors such as spinner flasks or rotational culture systems [29,33,46,47]. In spinner flasks, impellers stir the culture medium continuously, ensuring uniform nutrient supply to the suspended cells and preventing cell adhesion to the surface of the flask, resulting in spheroid formation [37]. In the case of rotational cell culture systems, the dynamic flow conditions are achieved using a rotating wall vessel containing the culture medium instead of using impellers or paddles [33]. Both the techniques employ specialized equipment to simulate a low-shear, low-turbulence environment with control over pH, temperature, and nutrient supply, as seen in vivo [32,48]. The techniques make possible high-throughput cell culturing with minimal involvement for extended periods of time [32]. However, controlling the motion of the suspension culture instead of the cells results in the high variability of the size and shape of the spheroids [32]. The dynamic conditions provide greater control over the shear stress applied on the suspended cells [49], and microcarriers are often employed in such systems to facilitate cell growth during proliferation [50,51]. Microcarriers are essentially tiny beads with sizes ranging from 100 to 300 μm, providing a large surface area for cell attachment by maintaining suspension in the dynamic culture medium [50].

Recent advancements in label-free cell culturing techniques have also popularized the use of acoustic-based and di-electrophoresis-based systems to culture cells in three dimensions without using scaffolds. Acoustic-based assembly employs non-invasive sound waves to assemble suspended cells in a culture medium, aggregating them to form spheroids [52]. Acoustic-based or acoustofluidic assembly is a contact-free and label-free approach utilizing bulk acoustic waves and Voronoi tessellation to cluster suspended cells; then, acoustic streaming is used to aggregate them into spheroids [53]. Acoustofluidic 3D cell culture systems integrate acoustic waves within microfluidic or disposable capillaries or devices to rapidly aggregate cells, forming uniform spheroids within minutes [53]. The di-electrophoresis-based system is another label-free technique that polarizes suspended cells in the culture medium through a non-uniform electric field, forming electric dipoles [52]. The cells are separated by a repulsive force, and the force subjected on the suspended cells is dependent on the cell size, allowing cells of identical sizes to aggregate together to form spheroids [54].

### 2.2. Scaffold-Based 3D Cell Cultures

The main principle behind the introduction of scaffolds in 3D cell cultures is to introduce an ECM-like structure that provides support for cell adhesion, the permeation of materials, proliferation, and migration. The biomaterials required for scaffold fabrication are identified based on a material’s biosafety, biocompatibility, biodegradability, and mechanical properties [55]. The nature of the cell type dictates the ECM architecture, requiring modifications of the scaffold in its nanoscale, microscale, and macroscale features to mimic the native tissue microenvironment [18]. The macroscale features regulate the overall size and shape, and the microscale features affect nutrient transport, while the nanoscale features dictate the cell response to and interaction with substrates [18]. Porosity is one of the primary traits of a scaffold facilitating cell adhesion, migration, and vascularization [56]. Porosity, pore distribution, and pore size dictate the nutrient exchange and, as a result, the overall cellular proliferation and differentiation [56]. 

**Figure 2:**
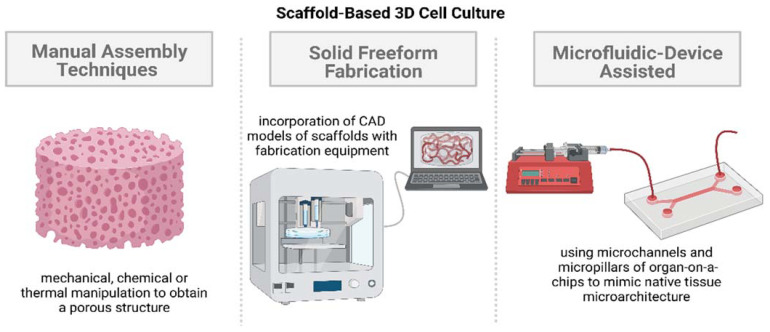
Illustration showing different approaches towards scaffold-based 3D cell culture models. Created with BioRender.com, accessed on 25 September 2022.

Since the scaffold features change with the cell type, several scaffolds and nutrient matrices have been developed over the years to tailor the porosity and surface characteristics [57]. Natural and synthetic biomaterials have both been employed in 3D cell culture techniques. Natural biomaterials showcase higher biocompatibility but suffer from batch-to-batch variations. Synthetic biomaterials, on the other hand, allow greater control over scaffold features to be obtained but suffer from the absence of bioactive characteristics [56]. The majority of the techniques developed over the years utilize hydrophilic cross-linked polymeric networks called hydrogels as the building materials for scaffolds or matrices. The major component of hydrogels is water, making their biophysical properties similar to those of native tissues [58,59]. Hydrogels can be either obtained from natural polymers or synthesized artificially, allowing control over the features needed to accurately mimic the ECM to be obtained, making them ideal biomaterials for scaffold fabrication. The different scaffold-based 3D cell culture approaches for accurate simulations of the native tissue microenvironment can be broadly categorized into three groups: manual assembly, solid freeform fabrication (SFF), and microfluidic-device-assisted systems (illustrated in Figure 2).

#### 2.2.1. Manual Assembly Techniques

Manual assembly techniques involve manual processes conducted using either the mechanical, thermal, or chemical manipulation of a material to create a porous scaffold. Manual assembly techniques focus on the surface chemical properties and biocompatibility of a biomaterial to mimic in vivo cell–cell and cell–ECM interactions. Common techniques for the manual fabrication of scaffolds include freeze-drying, gas foaming, phase separation, and solvent casting and particulate leaching, with freeze-drying being a widely used procedure for 3D cell cultures owing to its simplicity [60]. These techniques are deemed to be “manual”, since the final structure of the scaffold is determined directly by the user’s actions (such as the choice of temperature, reagent, etc.), instead of utilizing an automated system to replicate the native tissue microarchitecture. These techniques can modulate the generation of microstructural features to simulate the process conditions required for native tissue function but have limited control in replicating the intricate architectures of the ECM [18]. Relative to scaffold-free 3D cell culture systems and 2D cell monolayers, there is still greater control over parameters such as pore size, connectivity, and geometry, allowing more accurate drug screening and efficacy studies to be conducted by adopting effective mass transport and nutrient exchange [18]. Further details on the manual assembly techniques are as follows:

1. Freeze-drying involves the creation of porous scaffolds through a controlled solvent sublimation process. The scaffold is frozen, and the embedded solvent, typically water, undergoes sublimation, leaving behind a porous scaffold structure [60]. The pore sizes, porosity, pore distribution, and connectivity of the scaffold are mainly influenced by the cooling rate and sublimation rate, controlled by altering the temperature and pressure conditions [56,61]. Cells are then seeded onto the porous freeze-dried scaffold and cultured, allowing the biological activities to be analyzed to obtain endpoint measurements.
2. Gas foaming can be conducted in several ways, with the main principle involving the nucleation and growth of gas bubbles distributed throughout a polymer [62]. The conventional gas foaming technique involves the addition of a foaming agent, such as sodium bicarbonate, to a polymer in an acidic environment, producing an inert gas, such as carbon dioxide or nitrogen, at low or high pressure [62]. A porous scaffold is obtained as the dispersed gas is removed from the polymer, and cells are then seeded onto the structure and cultured to simulate the tissue microenvironment of interest. Gas foaming is a convenient technique for the fabrication of scaffolds with high porosity and interconnectivity [62]. However, the process has limited application due to the biocompatibility issues arising from the toxicity of the surfactant residue [62].
3. Phase separation methodologies are also widely accepted for generating scaffolds showcasing ideal biomechanical properties, high porosity, and interconnectivity. Thermally induced phase separation (TIPS) is a common approach to the fabrication of scaffolds with a hierarchical pore structure using composite polymer matrix or inorganic filler foams [63]. The main principle of this technique is the separation of a homogeneous polymer solution (solid–liquid or liquid–liquid polymer solvent solution) into a polymer-rich phase and a polymerless phase via a change in temperature [63]. The ability to optimize the process parameters, such as the choice of polymer, solvent composition, temperature control, coarsening process, and the incorporation of inorganic particles, provides close control in the final structure of the scaffold, allowing accurate in vivo tissue microenvironment replication to be conducted [64].
4. Another manual assembly scaffold-based cell culture approach that is popularly used to mimic bone marrow niche is the solvent-casting and particulate leaching technique (SCPL) [65]. The technique starts with the mixing of a polymer–solvent solution with an insoluble salt [65]. The mixture is heated to evaporate the solvent, leaving behind a salt–polymer composite, which is washed or submerged to leach out the salt to obtain a porous scaffold [65]. The SCPL technique is straightforward and does not require any special, expensive equipment to generate scaffolds with high porosity and interconnectivity but suffers from the scalability and limited bioactive properties of the resulting thin membranes [65,66].
5. Shape-changing and self-folding processes to form 3D tubular systems from initial 2D structures have also started to get employed in 3D cell culture systems [67]. Self-rollable elastomeric films can consist of key surface topographical patterns, making possible cell encapsulation and application as tissue building blocks [67,68]. Two-dimensional layers constructed from hydrogels give greater control and customization of key parameters such as degree of swelling, network pore size, crosslinking degree, and stiffness [67]. The technique also permits the co-culturing of multiple cell types in the resulting folded 3D scaffold structure to be performed, giving rise to a hierarchical organisation and internal microvascularization, allowing the accurate replication of native tissues to be performed [67]. However, the overall rolling process can result in physical deformation and requires significant efforts to ensure repeatable results in terms of the final shape of the scaffold.

#### 2.2.2. Solid Freeform Fabrication

Solid freeform fabrication (SFF) uses automated algorithms to fabricate a structure layer by layer based on a 3D model without involving molds. The application of solid freeform fabrication methodologies in 3D cell cultures addresses the limitations of manual assembly techniques in replicating the intricate architectures and morphologies present in the native tissue microenvironment. SFF utilizes computer-aided design (CAD), computed tomography (CT), or magnetic resonance imaging (MRI) data to create 3D models of scaffolds identical to the structure of the in vivo ECM [69]. In 3D cell culture studies, commonly used freeform fabrication approaches for generating biomimetic scaffolds are electrospinning, bioprinting, and selective laser sintering (SLS), detailed as follows:

1. Electrospinning (ES) involves the alteration of an electric field to fabricate continuous thin micro-/nanofibers from microspheres [70]. The conventional ES procedure consists of a solution reservoir connected to a nozzle, high-voltage direct current source, a flow rate controller, and a grounded collector [69,70]. A microsphere is formed at the nozzle tip due to the difference in potential between the nozzle tip and the grounded collector, which gets stretched due to the change in the electric field, forming a conical shape called the Taylor cone [70]. The electrostatic force creates a liquid jet, resulting in a randomly oriented fibrous mat [70]. These continuous fibers form a large surface area-to-volume ratio, making the scaffold ideal for cell attachment, proliferation, and differentiation [70]. Control over process parameters such as solution concentration, nozzle tip and grounded collector distance, and applied voltage allows of the scaffold features to be adjusted to replicate the natural ECM of interest [69].
2. Bioprinting is an additive manufacturing technique utilizing 3D printing principles to generate scaffolds using bioinks. Bioinks are synthesized based on the ECM characteristics of the native tissue of interest, consisting of biomaterials (hydrogels as base materials), active biomolecules, and even cells [71]. The ability to incorporate cells within the bioink allows the homogeneous distribution of cells in the scaffold structure to be obtained [71], making the process ideal for 3D cell culture studies. The scaffold structure is fabricated layer by layer based on the instructions outlined in the standard triangle language (STL) file obtained from the CAD model of the native ECM. The bioprinting of scaffolds can be conducted using three techniques: droplet-based, extrusion-based, or laser-based systems [71]. Laser-based systems, such as stereolithography, digital light processing, and two-photon polymerization bioprinting, utilize photo-crosslinking, allowing the accurate, controlled fabrication of in vivo-like vascular structures to be performed owing to the high resolution (≤20 μm [71]) compared with extrusion-based bioprinting. Each bioprinting technique has its own sets of advantages and disadvantages, but the ideal technique is chosen based on the tissue of interest and on whether the spatial resolution of the technique can accurately replicate the tissue microenvironment or not [71].
3. Multilayer scaffolds requiring substantial mechanical integrity can also be fabricated using a high-power laser-based system called selective laser sintering (SLS). SLS allows structurally complex scaffolds with controlled pore sizes, porosity, and topology to be fabricated [72]. The main principle of SLS is the fusion of powders (bio-ceramics) based on a CAD model of the scaffold. Conventional SLS is carried out using a carbon dioxide laser, which increases the temperature at the focal point, causing the powder to melt and fuse together [72]. Each layer is scanned by the laser, and the powder bed is lowered by one-layer thickness; the process is repeated to create a multilayer porous structure [72]. SLS has gained popularity over the years in bone tissue engineering, owing to the greater control in fabricating constructs with tunable mechanical properties, interconnected macropores and micropores to achieve vascularization, and sustaining a high density of cells [73].

#### 2.2.3. Microfluidic-Device-Assisted Systems

Although advances in microfabrication and SFF techniques allow the native ECM morphology to be replicated in scaffolds, the absence of fluid flow perfusion restricts the accurate simulation of the in vivo dynamic tissue microenvironment [8]. The incorporation of microfluidic devices in 3D cell culturing systems allows precise control of nutrient and drug delivery to be obtained, making them ideal platforms for drug screening studies. Microfluidic devices utilize microfabrication techniques to form microchannels and micropillars, providing a solid surface for cell attachment and proliferation to form a native-ECM-like physical environment [74]. The substrate used in the development of microfluidic devices can be categorized as either glass/silicon-based, polymer-based, or paper-based, with poly(dimethylsiloxane) (PDMS) being the most widely used material for microfluidic chip fabrication [74]. The microfabrication technique is dependent on the substrate used, including techniques such as photolithography, replica molding, injection molding, and 3D printing [8]. Unlike other 3D cell culturing techniques, microfluidic devices allow many different cell types to be cultured by altering the design of microchannels and micropillars. Microfluidic devices can be reused, and the small volumes of the microchannels reduce the costs associated with reagents in drug screening and efficacy studies [8]. Microfluidic devices make possible fluid flow through the inlets and outlets connected to the microchannels, and the incorporation of biosensors with the device permits the convenient execution of high-throughput drug screening studies [8]. However, the small volumes of the microchannels also limit the number of cells present in the device, requiring the need for expensive and specialized screening techniques [8].

## 3. Vascularization Strategies in 3D Cell Culture Models

Vasculature is formed by two main processes: vasculogenesis and angiogenesis [75]. Vasculogenesis refers to the de novo formation process observed when precursor cells such as angioblasts differentiate into endothelial cells, sparking the onset of the formation of a primitive vascular network [76]. Angiogenesis, however, refers to the development of newer vasculature from pre-existing blood vessels [77]. Angiogenesis is the preferred methodology to introduce vascularization in 3D tissue models; it is preferred over creating tubule-like vascular structures, partly due to the difficulty in the high-precision fabrication of capillary structures (<10 μm) [77]. Scaffold-free 3D tissue cultures typically involve the addition of angiogenic factors. In the case of scaffold-based models, on the other hand, the presence of a 3D architecture with tailored pore formation and interconnected pores promotes the formation of vasculature [55]. In both cases, the presence of endothelial cells (ECs), along with growth factors and ECM proteins, is essential for initiating angiogenesis and achieving vascularization [78]. Vascular endothelial growth factor induces angiogenesis by attracting ECs and facilitating EC proliferation, as well as promoting the formation of networked structures in the presence of angiopoietin-2 [78]. Mesenchymal stem cells are also observed to migrate towards the site of neovascularization owing to the presence of platelet-derived growth factor-BB (PDGF-BB) and angiopoietin-1 [78]. In this section, some of the recent studies in 3D cell culture models (2017–2022), summarized in Table 2, showcasing the presence or creation of vasculature are highlighted, particularly focusing on the underlying techniques involved in achieving vascularization.

### 3.1. Spheroid-Based 3D Cell Culture Models

**Figure 3:**
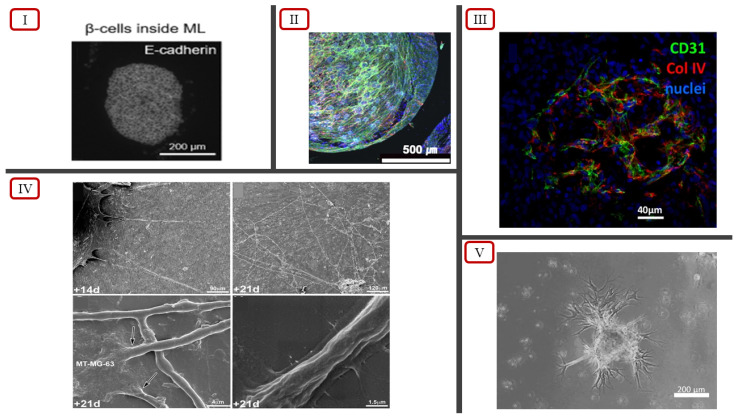
(**I**) Spatial distribution of β cells inside EC spheroid, reported by Urbanczyk et al. [83] (licensed under CC BY 4.0). (**II**) Actin cytoskeletal organization and pre-vascular patterns in MSC/HUVEC spheroids encapsulated in collagen/fibrin hydrogels, reported by Heo et al. [79]. (**III**) Basal membrane highlighted using anti-collagen IV staining in vascularized spheroids, reported by Muller et al. [81] (licensed under CC BY 4.0). (**IV**) Scanning electron microscopy showcasing formation of endothelial cell tubes (indicated by arrows), reported by Chaddad et al. [85]. (**V**) Sprouting branches of co-cultured spheroids, reported by Kim et al. [84].

In recent years, 3D spheroid models have employed co-culturing techniques using mesenchymal stem cells (MSCs) and human umbilical vein endothelial cells (HUVECs) to develop more accurate tissue models displaying vascularization. Co-culturing introduces heterocellular interactions, which are typically absent in monolayer stem cell cultures, in tissue models [79]. Heo et al. incorporated this concept to generate MSC-only and MSC/HUVEC spheroids in AggreWell plates to introduce a method to construct scalable bone tissue constructs [79]. The self-assembly tendency of cells in suspension and the cell-repellent property of the wells facilitated the aggregation process to form uniform 3D cellular spheroids [79]. Four different forms of cell cultures were conducted: MSC-suspension (Group No. 1), MSC/HUVEC-suspension (Group No. 2), MSC-only spheroids (Group No. 3), and MSC/HUVEC spheroids encapsulated in collagen/fibrin hydrogels (Group No. 4). Vascular network formation was observed in Group No. 4 throughout the hydrogel structure. Groups 2 and 4 showed positive results for cluster of differentiation 31 (CD31) expression, an endothelial cell (EC) marker used for detecting angiogenesis [79,80]. Group No. 4 showcased the highest actin cytoskeletal organization and pre-vascular network formation throughout the entire structure (Figure 3(II)), with the highest cell viability of 93.3% on Day 7, compared with the other groups [79]. This suggests that the incorporation of collagen/fibrin hydrogels along with MSC/HUVEC spheroids may be a simplistic approach towards achieving pre-vascular bone formation in 3D tissue models. Therefore, the enhanced functionality observed due to the presence of vascularized networks shows potential for the spheroid model to be used not only for cell physiology studies but also for disease modeling and drug screening.

Certain native tissues such as the subcutaneous adipose tissue (AT) are highly vascularized and are dependent on their vasculature to maintain proper functioning [81]. Muller et al. (2019) developed a modified forced floating cell culture approach using ULA to generate vascularized adipose spheroids from stromal–vascular fraction (SVF)-derived cells [81]. The research study employed a culture condition of using endothelial growth medium 2 (EGM2) instead of the standard culture of minimum essential medium α (αMEM) with 10% fetal calf serum (FCS) [81]. Culturing in pro-angiogenic EGM2 has been observed to facilitate the sprouting and vascularization process better than culturing in basal media alone [82]. The spheroids were encapsulated into Matrigel droplets 6 days after seeding and were cultured for 4 more days in an agitated medium via stirring. The results of the experiment showed the presence of dense and highly organized vascular networks throughout the spheroids, showcasing a morphology similar to that of in vivo vasculature (Figure 3(III)) [81]. Therefore, the vascularized adipose spheroid model can be used to accurately model human adipose tissue in vitro.

Conventional techniques of co-culturing cells in spheroids face issues in controlling the size of heterotypic spheroids due to spontaneous aggregation, especially in the case of native tissue with dense vascularization [83]. Urbanczyk et al. tackled this issue by conducting one of the first studies where co-cultured β cells and ECs showed successful stimulation and functionality of β cells using the magnetic levitation method [83]. Using a low-adhesion 96-well plate, the researchers conducted three groups of 3D cell cultures using magnetic levitation: a 1:1 even distribution of β cells and HUVECs, β cells encased by HUVECs, and HUVECs encased by β cells, based on naturally occurring spatial distributions as observed in native pancreatic tissue. The results of the cultures were compared to those of cultures utilizing spontaneous aggregation instead of magnetic levitation and showcased heterogenous distribution along with distinguishable expression of CD31. The functionalities of the cell culture groups were assessed with insulin secretion, where the constructs with β cells inside the HUVECs showcased the highest insulin secretory function compared with the other cell culture groups [83]. Therefore, this in vitro model makes possible further development towards achieving prevascularization in transplantable islet grafts. 

Another technique to eliminate the issue of the distribution of multiple cell types in heterotypic spheroids during co-culturing is using cell sheet engineering [43]. Kim et al. reported a modified cell sheet engineering approach where they harvested uniformly sized heterocellular spheroids from a micro cell sheet (µCS) assembly rather than using a suspension culture [84]. A micro-patterned hydrogel surface was obtained through conformal contact from a PDMS stamp, where human turbinate-derived MSCs (hTMSCs) and adipose-derived stem cells (ADSCs) were seeded and incubated under normal culture conditions [84]. The seeding was conducted in different ways: Group S involved seeding HUVECs 24 h after the initial seeding of hTMSCs or ADSCs (core shell), while Group M involved the direct seeding of stem cells and HUVECs. Harvested spheroids from the two groups were fused and demonstrated the presence of significant network structure in S, with limited network formation observed in M. The final results of the experiment showed a six-fold increase in the CD31 positive area for spheroids harvested from the core-shell structure of both the cell types, advocating the use of this technique to generate spheroids to be used as “building blocks” for constructing 3D complex micro-tissues [84]. The investigation also highlighted how the positioning of the endothelial cells plays a crucial role in the vascularization process compared with solely having a heterotypic spheroid model.

Although the spheroid models have limitations in terms of size, scalability, and architecture, spheroids are still considered to be the best in vitro models for investigating drug sensitivity and resistance [85]. Chaddad et al. created a novel approach by incorporating a 2D monolayer culture together with spheroids, creating a combination 2D/3D model to better replicate the in vivo tumor microenvironment [85]. The 2D cell monolayer was formed by culturing HUVECs in an endothelial cell growth medium, and the spheroids were generated from the 3D culture of osteosarcoma cells (MG-63) using the hanging drop technique [85]. The initial direct contact between the MG-63 spheroids and the HUVEC monolayer resulted in the death of HUVEC cells. However, the direct contact also influenced the cancer cells to have a significant increase in the secretion of the angiogenic factor called vascular endothelial growth factor (VEGF). The increased VEGF expression resulted in the formation of tubule-like structure and lumen (Figure 3(IV)), only at locations above and under the tumor cells, interpenetrating and tangling around them [85]. The results of this study further validate the importance and necessity of having a 3D environment, allowing endothelial cell migration to form the tubular networks [85]. Although 2D cell monolayers are unable to accurately portray the native tissue microenvironment, the incorporation of 3D spheroids with 2D systems results in the creation of an accurate tissue model, owing to the enhanced vascularization.

### 3.2. Bioprinting-Based 3D Cell Culture Models

**Figure 4:**
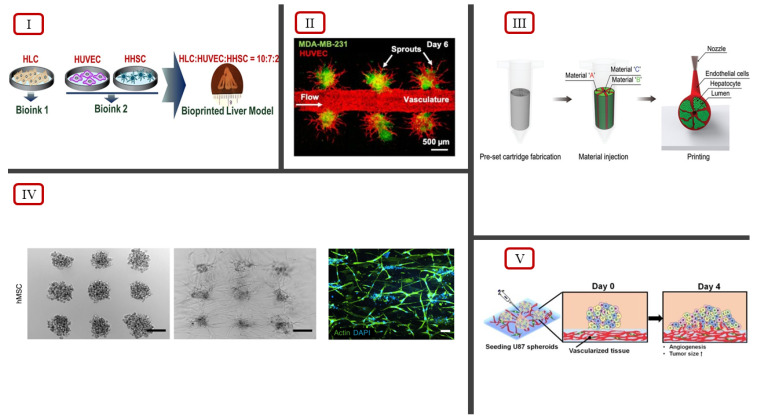
(**I**) Bioink ratio to bioprint hepatic liver lobule model, illustrated by Janani et al. [88]. (**II**) Tumor angiogenesis observed using fluorescent imaging, reported by Dey et al. [90]. (**III**) Preset extrusion bioprinting technique to model hepatic lobule structure, illustrated by Kang et al. [89]. (**IV**) Self-organization of hMSCs via extrusion bioprinting using the BATE concept, reported by Brassard et al., scale bar 500 μm [87]. (**V**) Illustration of angiogenesis of MCTSs seeded on bioprinted vascularized tissue, reported by Han et al. [86] (licensed under CC BY 4.0).

The combination of 2D cell monolayers with MG-63 spheroid models showcases an improved vascularization approach to 3D cell culturing systems [85]. However, the challenge remains on the fact that the proposed technique may not be able to accurately measure drug response in the combined culture compared with the in vivo tumor microenvironment. Han et al. explored a similar combination approach to investigate the in vitro drug testing capabilities, utilizing the seeding of multicellular tumor spheroids in a bioprinted blood vessel layer instead of a 2D cell monolayer [86]. Using a cell-ladened bioink consisting of HUVECs and lung fibroblasts (LFs) in gelatin/alginate/fibrinogen (GAF) hydrogel, a blood vessel layer was bioprinted using an extrusion-based system. After confirming the presence of a vascularized structure in the blood vessel layer, uniformly sized multicellular tumor spheroids (MCTSs) were seeded onto the blood vessel layer and incubated until the migration of the networked structure into the MCTSs was observed (Figure 4(V)). A control group of a GAF layer without vascularized tissues was also seeded with MCTSs and incubated. The infiltration of the networked structure into the MCTSs was only observed with the vascularized tissue, indicating the importance of blood vessels and fibroblasts in proliferation, angiogenesis, and migration [86]. The neovascularized structure also showed disorganized blood vessel networks, a key characteristic of tumor microenvironments due to induced micro-regional hypoxia from limited oxygen supply. The effects of the combined treatment of anti-cancer drug temozolomide (TMZ) and blood vessel inhibitor sunitinib (SU) on the biofabricated structure were investigated to validate the feasibility of using this approach for in vitro studies. The results of the investigation were coherent with the results of combination therapy involving TMZ and SU with U87 cells in mice. Therefore, this approach produces reliable in vitro models for testing anti-cancer drugs due to an accurate replication of the in vivo tumor microenvironment.

Brassard et al. addressed the current limitations of bioprinting technologies to reproduce intricate micro-architectures, cell-type diversity, and native tissue functionality using a bioprinting-assisted tissue emergence (BATE) approach [87]. The approach influences stem cells and organoids to fuse and reorganize through the geometry and constraints of the 3D printing process, making them act as self-organizing building blocks. The bioprinter consists of a syringe-based extrusion system coupled with a microscope, providing real-time feedback to the user to control and modulate the printing process. Manual adjustments on the nozzle size, flow rate, and printing speed dictate the final cellular density, allowing the extrusion of lines of single cells to be conducted. The multicellular self-organization behavior allows epithelial tubes, connective tissues, and vascular networks to be printed with a defined geometry (Figure 4(IV)). This study illustrated that the use of key cell types of the native tissue as self-organizing building blocks allows the replication of the intricate ECM and tissue architecture to be performed, reproducing the tissue–tissue interactions as observed in the native tissue microenvironment.

Drug toxicity and efficacy studies for densely vascularized multicellular native tissues have become possible with the incorporation of bioprinting in 3D cell culture models. Bioprinting allows native ECM architectures to be recreated by fabricating constructs with the accurate spatial distribution of multiple cell types [88]. Janani et al. demonstrated the successful bioprinting of a 3D model replicating the native liver lobule microarchitecture using a dual-extrusion head bioprinter [88]. A CAD model depicting the 3D liver lobule structure was used to print six layers of cell-ladened hydrogels. Each extrusion head consisted of its own bioink encapsulated in hydrogel layers; parenchymal bioink 1 was constituted of stem cell-derived hepatocyte-like cells (HLCs), and non-parenchymal bioink 2 was constituted of HUVECs and human hepatic stellate cells (HHSCs). Each bioprinted layer consisted of three alternative multimaterial extrusions of bioinks 1 and 2 in order to replicate the complementary arrangement in the native liver (Figure 4(I)). Live/dead confocal fluorescence images confirmed vascularization. as the lateral sections of HLCs/HUVECs/HHSCs showed uniform cellular distribution, cell viability, and proliferation. Drug screening studies were conducted with the addition of non-hepatotoxicants (aspirin and dexamethasone), an idiosyncratic hepatotoxicant (trovafloxacin mesylate), and known hepatotoxicants (acetaminophen and troglitazone). The results of the toxicity studies showed that the model was more sensitive than the existing reported results. The absence of primary human hepatocytes, Kupffer cells, and bile epithelium, as well as the lack of a continuous perfusion system, might have caused the discrepancies observed in the drug toxicity results. The bioprinted liver model did exhibit similar physiological and metabolic properties, requiring the need to conduct a follow-up to create a more accurate, biomimetic model of native liver tissue.

The discrepancy in the results of in vitro liver tissue models is due to the difficulties associated with culturing primary hepatocytes in both 2D and 3D systems, even though hepatocytes make up 80% of total liver volume [89]. Using an immortalized human hepatocellular carcinoma cell line, HepG2/C3A, Kang et al. successfully bioprinted a hepatic lobule structure with a preset extrusion bioprinting technique [89]. The bioprinting technique involved extrusion through a preset cartridge mimicking the structure of a human lobule (Figure 4(III)). The design of the cartridge consisted of three separate segments for separating the hepatocyte-laden bioink, EC-laden bioink, and cell-free bioink as sacrificial material. The bioprinted structure showcased the spatial arrangement of endothelial cells and hepatocytes, and an interconnected central lumen, replicating cellular organization within a sinusoid network of a hepatic lobule. Vascularization was achieved by incubating the bioprinted structure at 37 °C to remove the alginate in the sacrificial bioink. The removal of alginate resulted in the formation of microchannels, allowing ECs to interconnect between the lumen and the exterior. The hepatic lobule structure also showed significant CD31 expression, further validating the interconnections between ECs and the presence of vascularization. Hepatotoxicity studies were conducted with the administration of amiodarone, an antiarrhythmic medication, to investigate the hepatic function and drug screening capabilities of the bioprinted structure. The investigation indicated increased drug resistance in the hepatic lobule structure compared with 2D models, with 2D models having a cell viability lower than 50% for low-dose amiodarone administration. Therefore, a preset cartridge extrusion-based bioprinting approach can result in the fabrication of hepatic lobule models, showcasing the stable secretion of albumin and urea, higher protein levels of albumin, multidrug resistance-associated protein 2 (MPR2), and CD31 relative to non-engineered models [89]. Adjustments in the bioprinting technique to include primary hepatocytes can be used to obtain accurate drug responses, making the hepatic lobule model a reliable 3D culture system for preclinical studies.

However, even with the fabrication of a biomimetic 3D architecture, the lack of fluid flow perfusion limits 3D cell culturing models in the accurate simulation of the dynamic native tissue microenvironment. Dey et al. addressed this limitation by devising a novel dynamically flow-based 3D vascularized breast tumor model with drug screening capabilities [90]. The model involved the aspiration-assisted bioprinting of heterotypic tumor spheroids, a composite collagen/fibrin-based matrix (C2F3) as the bioprinting substrate, central vasculature, and a perfusion chamber with connection ports. Heterotypic tumor spheroids were generated using forced floating principles by culturing HUVECs, metastatic breast cancer cells (MDA-MB-231), and fibroblasts (HDFs) in a U-bottom cell-repellant plate. The central vasculature was formed using the pin-molding technique. A steel wire was inserted through the connection ports of the perfusion chamber, and the HDF-laden C2F3 hydrogel was added and was allowed to crosslink. HUVECs were introduced to the open channel after the removal of the steel wire, resulting in central endothelialized vasculature. Aspiration-assisted bioprinting was employed to ensure that the positioning of tumor spheroids from the central vasculature was precisely controlled. Significant angiogenesis was observed in tumor spheroids in close proximity to the central vasculature (~100 µm) with increased vessel length, branching, and sprouting density, compared with distal spheroids (~500 µm) (Figure 4(II)). Doxorubicin, an anthracycline-based chemotherapeutic drug, was administered to validate the drug screening capabilities of the model. The results of the experiment successfully demonstrated a dose-dependent reduction in tumor volume following doxorubicin uptake [90]. HER2-targeting chimeric antigen receptor (CAR)-modified CD8+ T cells were also perfused through the central vasculature, and significant T-cell infiltration was observed in the tumors, along with a reduction in tumor growth. The novel device developed by Dey et al. showcased successful perfusion, angiogenesis, and dynamic flow, demonstrating the potential for a physiologically relevant drug and immunotherapy screening platform.

### 3.3. Microfluidic-Device-Based 3D Cell Culture Models

Having centralized vasculature allowing the dynamic flow of nutrients to be obtained in a 3D cell culture system limits the cell proliferation region to only areas surrounding the vasculature. As a result, angiogenic sprouting is only observed in singular spheroid structures lacking interconnection within the spheroid model, as observed in Figure 4(II). Incorporating microfluidic devices in 3D cell culture models provides a method to introduce the dynamic flow conditions throughout the entirety of the structure, allowing the accurate recapitulation of the native tissue microenvironment to be performed.

Sano et al. engineered a method to introduce perfusable vascular networks within multicellular spheroids using a PDMS microfluidic chip [91]. A hydrogel was embedded within the microfluidic device where ECs were co-cultured with lung fibroblasts (LFs) to form vascular networks connected to the microchannels of the device. Multicellular spheroids were prepared using HUVECs, LFs, and either MSCs or osteo-differentiated MSCs, which were seeded into the fibrin–collagen gel on both sides of the central microchannel. LFs were added to facilitate the angiogenesis process, while HUVECs could form interconnected networks between the spheroids and microchannels. The angiogenic sprouting from within the spheroids anastomosed to the microchannels was observed, confirming the vascularization of the spheroids. FITC-dextran solution was added inside a side-microchannel, and fluorescence imaging confirmed FITC-dextran solution flow without leakage, validating that the vascular networks were perfusable. The microfluidic chip was investigated for modeling cancer metastasis by having one side of the microchannel showcase a bone-mimicking microenvironment using osteo-spheroids. The increased migration of MDA-MB-231 breast cancer cells towards the side of the bone-mimicking microchannel showcased the capability of this 3D cell culturing system to model cancer metastasis. Therefore, the incorporation of microfluidic chips with conventional 3D cell culturing offers a new approach towards the vascularization of multicellular spheroids and the creation of an in vivo-like tissue microenvironment.

**Figure 5:**
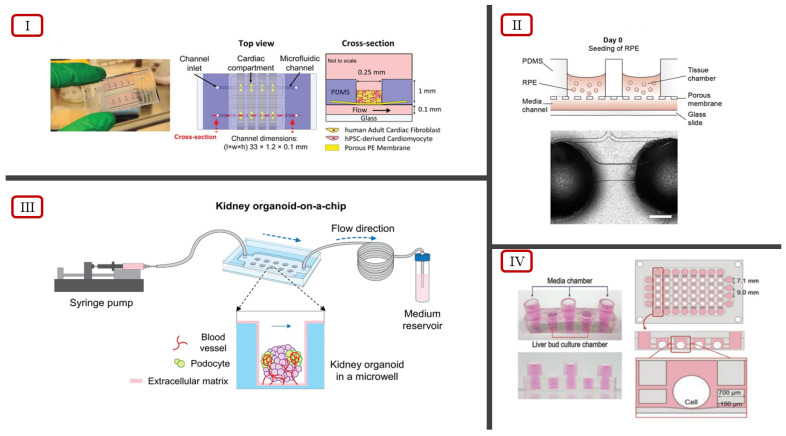
(**I**) Heart-on-a-chip device, illustration of the multilayer model by Vivas et al. [94]. (**II**) Retina-on-a-chip device (top) and imaging showcasing ROC seeding into device, reported by Achberger et al., scale bar 500 μm [93] (licensed under CC BY 4.0). (**III**) Kidney-on-a-chip device, illustration of the dynamic culture system by Lee et al. [92] (licensed under CC BY 4.0). (**IV**) Microfluidic system for generating liver organoids (left) and multiorgan model (right), reported by Jin et al. [95].

Although 3D cell culture systems have started employing dynamic flow conditions to better recapitulate the interactions in the native tissue microenvironment, vascularization is mainly achieved under static culture conditions [92]. Lee et at. developed a kidney organoid-on-a-chip system, investigating the effects of shear stress from dynamic fluid flow on kidney organoids [92]. Kidney organoids used for drug screening systems are commonly generated from human pluripotent stem cells (hPSCs) under static culture conditions [92]. The generated organoids lack vascularization, limiting growth and maturation, making them anatomically different from native kidney tissues. The kidney organoid-on-a-chip system utilized a PDMS chip, and the kidney organoids were cultured in compartmentalized microwells (Figure 5(III)). The optimal dimensions of the microwells were obtained from computational simulations of the laminar flow and the analysis of the shear stress associated with the fluidic flow. A control group of kidney organoids was cultured under static conditions to compare the vascularization and maturation of the cultured organoids. The results of the investigation showed that kidney organoids cultured under static conditions had limited vasculature and tubular epithelium, while kidney organoids cultured under fluid flow conditions showed enhanced vascularization and foot process maturation [92]. Drug toxicity investigation using different concentrations of tacrolimus, a nephrotoxic drug, was conducted on organoids either cultured under static or dynamic flow conditions. The results of the investigation showed increased sensitivity in the organoids cultured under dynamic flow conditions compared with the static culture, highlighting the drug screening potential of the system. The kidney organoid-on-a-chip system reveals the necessity to incorporate all the different stimuli present in the native tissue microenvironment to allow accurate replication in 3D cell culture systems to be obtained.

Conventional 3D cell culturing systems depend on the formation of interconnected networks or micropores to establish a perfusable vascularized model. Microfluidic-based approaches eliminate the need for tubule-like structure formation by allowing precise control in fabricating vasculature-like perfusable microchannels to be obtained. Achberger et al. incorporated the principles of microfluidic chips to develop the retina-on-a-chip (ROC) model, a vascularized 3D in vitro model of the human retina [93]. Retinal organoids (ROs) derived from human embryonic stem cells or human induced pluripotent stem cells have limited modeling capabilities of the in vivo conditions observed in human retina due to the lack of vasculature. Using a layered microfluidic chip, ROC achieved vasculature-like perfusion by connecting four identical micro-tissues via microchannels on a top-layer and a bottom layer with a channel for controlled fluid flow (Figure 5(II)). A thin, porous membrane mimicking the endothelial barrier separated the two layers, sheltered against shear forces, and ensured material exchange between the layers. The ROC model was the first in vitro system to replicate key in vivo physiological features by showcasing interactions between photoreceptors and the retinal pigment epithelium. Drug screening investigations using chloroquine and gentamicin provided the accurate replication of the side-effects of chloroquine and the mimicry of gentamicin-induced retinopathy [93]. Therefore, the introduction of vasculature-like perfusion using microfluidic systems allowed the accurate replication of the in vivo characteristics to be performed and validated ROC as a potential drug screening tool.

The accurate replication of certain native tissue microenvironments requires the introduction of external stimuli in 3D cell culture models. As a result, microfluidic-based systems are often incorporated in tissue models owing to the ease of fabrication and compatibility of integrating external parameters. Vivas et al. developed a novel bi-compartmental, monolithic heart-on-a-chip device, capable of integrating 3D carbon electrodes for electrical pacing and the culture of cardiac tissue [94]. Vascularization was introduced via the active perfusion between the two microcompartments separated by a semi-permeable membrane. Each layer accommodated a certain tissue type of either cardiac tissues or endothelial cells, making possible dynamic fluid flow through the endothelial layer without risking shear stress on the cardiac tissues. The device featured an open-top compartment in the top layer, allowing cardiac cell suspensions, medium sampling, and cardiac microtissue retrieval to be obtained, and an electronic lid was introduced for cardiac pacing (Figure 5(I)) [94]. Electromechanical stimulations, the presence of an endothelial monolayer, and selective perfusion promoted the maturation of hPSC-derived cardiomyocytes for the accurate recapitulation of the cardiac tissue microenvironment. The heart-on-a-chip model provides an approach for modeling cardiac physiology via heterotypic cell interaction, electrical actuation, and biochemical cues [94]. 

In addition to its own cell–cell and cell–ECM interactions, the native tissue microenvironment is also influenced by the interactions of different tissues nearby. Jin et al. developed a microfluidic system for generating 3D vascularized liver organoids for high-throughput drug screening and conducted multiorgan model studies (Figure 5(IV)) [95]. The approach addressed the difficulty in culturing hepatocytes using the Transcription factor (TF)-mediated direct reprogramming of iPSCs to form induced hepatic (iHep) cells. The densely vascularized liver-specific microenvironment was mimicked using decellularized liver extracellular matrix (LEM) hydrogel in the microfluidic device. Vascularization was achieved in the liver organoid by co-culturing of iHep cells and HUVECs in a 3D LEM hydrogel reconstituted in the microfluidic device [95]. The culture was subjected to dynamic flow conditions using a rocker system utilizing gravity instead of pumps. The immunostaining results showed an increased presence of albumin in the organoids cultured under dynamic flow compared with static, indicating the vascularization of the organoids. Drug screening potential was explored by investigating the response of the liver organoids in the microfluidic chips to different drugs. The results of the investigation indicated higher sensitivity to drug testing, and a more mature and functional hepatic state. An array format of the microfluidic device was used to design a multiorgan model to check the feasibility of the model as a high-throughput drug testing and screening platform. Three-dimensional micro-hepatic tissue (iHE-F) was cultured alongside small intestinal and stomach organoids using a tripartite culture system, generating a multiorgan model. Each of the organoids showcased its own linear specific markers, demonstrated functionality, and valid crosstalk among organoids, validating the high-throughput testing potential of the multiorgan model. The results indicated that the 3D hepatic cell culture model utilized microfluidic principles to create an accurate drug testing and screening platform for liver organoids, indicating it to be a multiorgan model capable of high-throughput screening.

**Table 2:** Summarized details of vascularization strategies in 3D cell culture models, starting from simplistic approaches using spheroid-based models to increasing complexities involving bioprinting-based models, to lastly, complex models utilizing microfluidic devices to recapitulate the native tissue microenvironment.

Three-Dimensional Cell Culture Model	Vascularization Approach	Highlights	References
Spheroid-based	Incorporation of collagen/fibrin hydrogels with MSC/HUVEC spheroids	Enhanced functionality due to the presence of vascularized networks	[79]
	Culturing SVF-derived cells in EGM2 using forced floating cell culture method	Presence of dense and highly organized vascular networks, showcasing morphology similar to that of in vivo vasculature	[81]
	Co-culturing β cells and ECs using magnetic levitation method	Heterogeneous distribution of cells, distinguishable CD31 expression, and significant stimulation of basal insulin secretion	[83]
	Seeding HUVECs, hTMSCs, and ADSCs on a micro-patterned hydrogel surface	Six-fold increase in CD31 expression for harvested spheroids, allowing them to be used as building blocks for constructing complex 3D microtissues	[84]
	Incorporating 2D cell monolayer of HUVECs with MG-63 spheroids cultured using hanging drop technique	Enhanced vascularization from increased VEGF expression	[85]
Bioprinting-based	Seeding MCTSs on bioprinted blood vessel layer using a cell-ladened bioink with HUVECs and LFs in GAF hydrogel	Significant vascularization and accurate anti-cancer drug treatment results; coherent with results in mice cells	[86]
	Fusing stem cells and organoids through bioprinting constraints, making them self-organizing building blocks	Ability to showcase multicellular self-organization and control over printing parameters to replicate native ECM	[87]
	Dual extrusion head bioprinter, utilizing two bioinks: parenchymal bioink 1 and non-parenchymal bioink 2	Exhibited similar physiological and metabolic properties, as well as highlighted the need of using primary cell lines for accurate modelling	[88]
	Preset extrusion bioprinting, where preset cartridge mimics the structure of a human hepatic lobule	Increased drug resistance and higher levels of albumin, MPR2, and CD31 relative to non-engineered models	[89]
	Dynamic flow-based 3D vascularized tumor model consisting of central vasculature and perfusion chamber	Significant angiogenesis and successful perfusion for a physiologically relevant drug and immunotherapy screening platform	[90]
Microfluidic-device-based	Multicellular spheroids using ECs and LFs seeded into fibrin–collagen hydrogel embedded within a microfluidic device	Showcased increased cellular migration, allowing accurate modeling and the studying of cancer metastasis to be performed	[91]
	Kidney organoid on-a-chip system involving a PDMS chip and organoids cultured in microwells under dynamic flow conditions	Cultured organoids showed increased vascularization and maturation	[92]
	Retina on-a-chip system involving a layered microfluidic chip and perfusion through connections via microchannels	First in vitro system to replicate key in vivo physiological features by showcasing interactions between photoreceptors and retinal pigment epithelium	[93]
	Bi-compartmental, monolithic heart-on-a-chip device capable of 3D carbon electrodes integration for electrical pacing	Accurate recapitulation of native cardiac tissue via electromechanical stimulations, endothelial monolayer, and selective perfusion	[94]
	Mimicking native ECM utilizing a decellularized liver ECM hydrogel in a microfluidic device	Increased drug sensitivity, and mature and functional hepatic state; high-throughput drug screening platform, and capable of multiorgan model studies	[95]

## 4. Concluding Remarks and Future Perspectives

Over the years, it has become evident the need of 3D models due to the lack of capability to mimic the in vivo environment with 2D cell monolayers. The simplest 3D cell culture models, even without the presence of vascularization, exhibit features and interactions similar to those of the native tissue microenvironment compared with 2D cell culture systems [27]. Simply identifying a culture medium to replicate the biomechanical and biochemical cues of the in vivo ECM and employing the self-aggregation tendency of cells can generate spheroid models that can provide a better physiological model for cell studies than 2D cultures. Vascularization, on the other hand, allows the scalability of the spheroid model to be performed by allowing sufficient nutrient supply to be provided and introduces the missing cell–ECM interactions from the development of basement membranes and interconnected networks. Adding these interactions is key to ensuring the proper maturation of cells, resulting in the accurate recreation of the native tissue model. In addition, vascularized 3D cell culture models also provide the opportunity to accurately simulate the cellular response to different drugs by allowing perfusion through the entire structure to be obtained, taking one step further from just the accumulation and penetration of drugs at the spheroid surface. Therefore, it is clear that achieving vascularization must be considered as a key factor in replicating the native tissue microenvironment when modeling 3D cell culture systems to conduct physiological or drug screening studies.

Vascularization is mainly achieved through the incorporation of endothelial cells, popularly HUVECs, with tissue-specific cells in a culture medium containing angiogenic growth factors. Recent developments in vascularization in spheroid models employ the co-culturing of HUVECs and checking for CD31 and VEGF expression. The cell type involved in the co-culture dictates the extent of vascularization, with cancer cells displaying increased formation of tubular networks compared with other cell types. Multicellular spheroids are increasingly used in order to recapitulate the heterotypic native tissue microenvironment. In addition to coculturing, optimizing parameters such as cell ratios, seeding density, culture medium, and coculturing time play vital roles in achieving sufficient in vitro prevascularization [96]. However, spheroid models suffer from the vascularization of singular units of spheroids, lacking interconnectivity and formation of the in vivo-like ECM. Even multicellular models have limited applicability due to the widespread use of HUVECs for endothelium modelling owing to ease of preparation, preventing the establishment of organ-specific models [96]. These limitations of spheroid models have been addressed with recent developments incorporating bioprinting and 3D cell culture systems.

Bioprinting allows the intricate ECM and tissue microarchitecture to be accurately replicated through a precise layer-by-layer fabrication process. Incorporating the spheroids in the bioink and the use of multiple bioinks allow the accurate spatial distribution of multiple cell types to be obtained, recreating the native ECM architecture required for ensuring proper vascularization. Sacrificial materials are also employed in bioinks, allowing densely vascularized constructs to be created through the formation of cavities or micropores. Challenges still remain in fabricating tubular constructs via bioprinting due to the poor resolution of extrusion-based systems. While laser-based bioprinting systems can satisfy the geometrical requirements, the fabrication conditions are often cytotoxic and result in decreased cell viability. In addition, bioprinting and spheroids are also unable to capture the dynamic flow conditions observed in vivo. Recent developments in microfluidic technologies have established microfluidic chips as the upcoming platform for 3D cell culturing and drug screening studies. Microfluidic devices make the following possible: the in vivo fluid flow conditions, vasculature-like perfusion without the need for tubular constructs, and easy integration into external systems for recreating the different stimuli observed in the native tissue microenvironment. 

The recent advancements in 3D cell culture systems highlight many techniques for achieving successful vascularization making drug screening and toxicity studies possible. Unfortunately, the lack of standardized protocols prevents the widespread use of 3D cell culture systems as drug screening platforms. In addition, challenges in the large-scale expansion of human cells create a bottleneck in scaling up 3D systems for regenerative medicine research [96]. Difficulties in producing commercially available assay formats and the reproducibility of complicated procedures also add challenges to the creation of standardized protocols [28]. The variations in the literature studies investigating drug interactions, cell differentiation, and cell signaling weaken the claims of standardization of protocols due to the limited repeatability of the results. The discrepancies observed in the investigations can be attributed to the inclination of researchers towards endpoint measurements instead of ensuring an accurate replication of the native tissue environment in 3D cell culture models. Three-dimensional cell culture models are also often viewed as isolated systems, disregarding the key interactions observed among different types of tissues in vivo. Observing a microarchitecture similar to that of the native tissue, the presence of sprouting, and/or the functionality of tissues are not necessarily sufficient to conclude that the in vitro model is identical to the in vivo tissue. Accurate, repeatable, and reproducible drug responses can only be obtained when the cell culture models not only include the primary cell types and the tissue microarchitecture but also consider the different biochemical and mechanical stimuli, surrounding cells, and the microenvironment influencing the native tissue in vivo. Advancements in technology have allowed state-of-the-art equipment to be employed, resulting in many novel approaches towards cell cultures. However, it seems that the advancements in technology may be a curse in disguise, as there are clear gaps in theories explaining the different phenomena observed at the cellular level. This strongly indicates that vascularization efforts in 3D cell cultures should ideally be geared towards models unraveling the complexities of the mechanism rather than checking for complete perfusion. Once the results for such models establish theories to bridge the gap in the current understanding of vascularization, the creation of drug screening platforms will likely become far less challenging.

## Data Availability

Not applicable.
